# Supplementary material for: Novel Modification of a Confirmatory SMA Sequencing Assay that Can Be Used to Determine SMN2 Copy Number
Source: Int J Neonatal Screen. 2021 Jul 21;7(3):47. doi: 10.3390/ijns7030047 (PMC8395917; doi:10.3390/ijns7030047)
Supplement: Supplementary file 1 [file IJNS-07-00047-s001.zip › IJNS-1223296-supplementary.pdf]

Supplementary Materials:

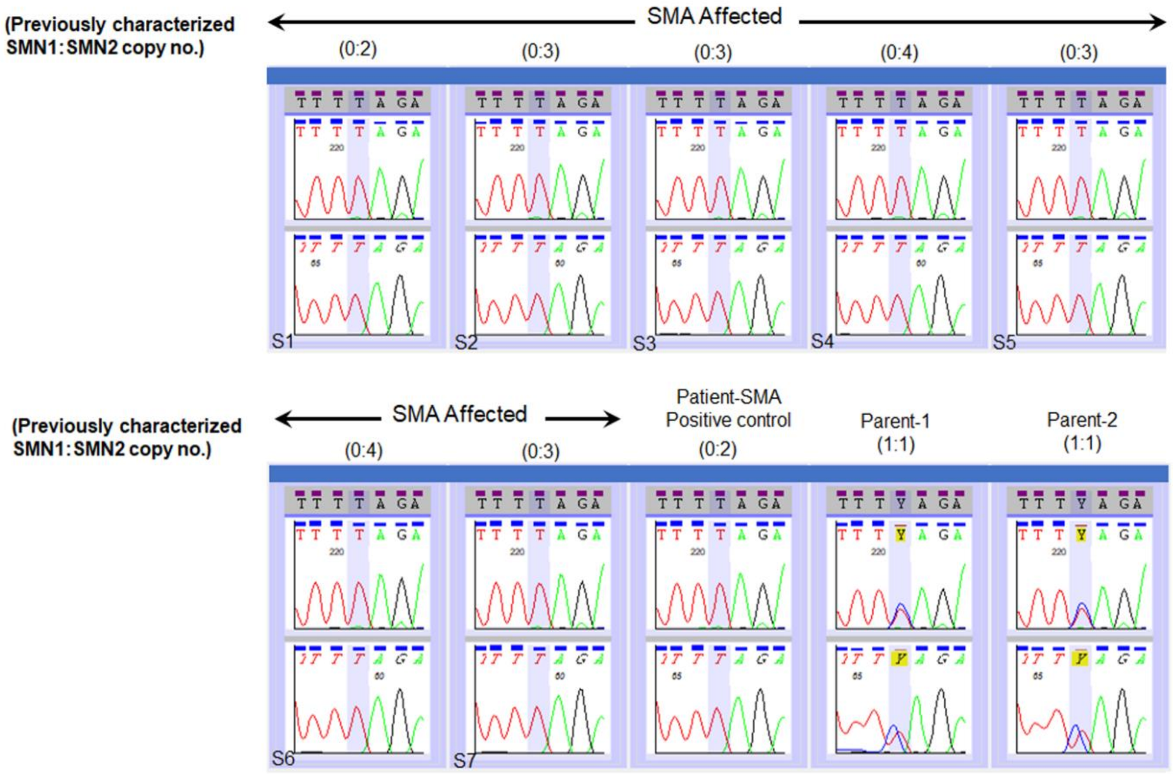

Figure S1: Genotype confirmation on the specimens subjected for *SMN2* copy validation. Specimens subjected to the validation study for *SMN2* copy were first confirmed for their genotype using Sanger sequencing as described in methods.

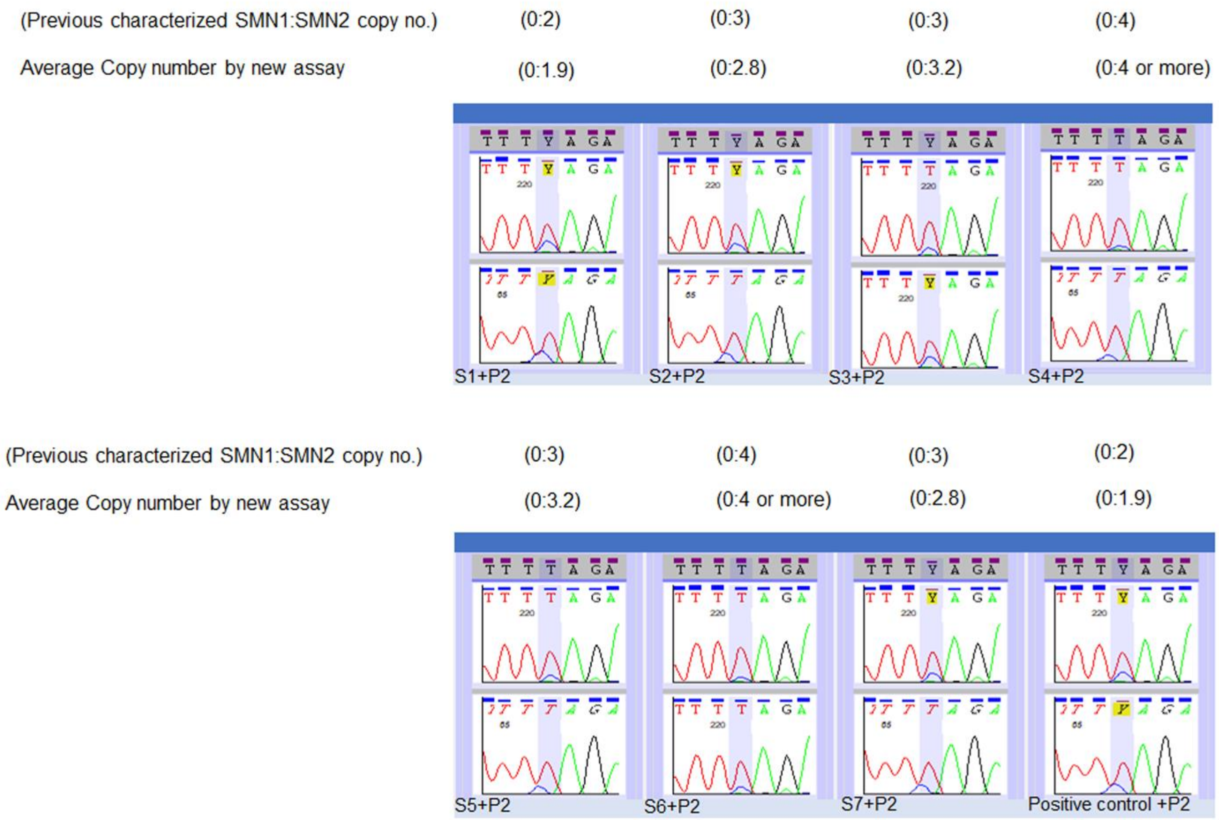

Figure S2: Determination of *SMN2* copy number assay validation using SMA affected specimens using another spike DNA (parent 2). DNA from SMA affected specimens and a Coriell DNA (positive control) were mixed in equal concentration with spike DNA, and bidirectional sequencing was performed. *SMN1* gene sequence used as reference sequence, and data were analyzed for determine the *SMN2* copy number in the test specimens as described in the methods section.
